# “What Is Normal?”: A Qualitative Exploration of Health Practitioners’ Reports of Treating Patients Presenting with Unpleasant Sexual Experiences

**DOI:** 10.1007/s10508-024-02994-3

**Published:** 2024-09-23

**Authors:** Rachael Sharman, Andrew Allen, Kirstyn van Niekerk, Alexandra Coles, Ramesh Manocha, Therese Foran

**Affiliations:** 1 School of Health, University of the Sunshine Coast, Sunshine Coast, 4556 Australia; 2 Health Ed, Sydney, Australia; 3 Faculty of Medicine and Health, University of Sydney, Sydney, Australia; 4 School of Clinical Medicine, University of New South Wales, Sydney, Australia

**Keywords:** Sexual health, Health practitioners, Sexual experiences, Unpleasant sex

## Abstract

Sexual health, including sexual pleasure, is fundamental to holistic health and well-being, and is considered an area of priority health in Australia. Despite the importance of sexual functioning, women experience significant gaps in sexual well-being compared to men and often do not seek medical care or treatment. Health practitioners are central to the identification and treatment of sexual dysfunction, including fostering sexual well-being for patients. Despite this, minimal research has explored health practitioners’ experiences in treating reports of unpleasant sex. This study aimed to explore health practitioners’ experiences, responses, and confidence in treating patients presenting for unpleasant sexual experiences. An online, mixed-methods survey was completed by 96 participants. Thematic analysis identified 11 core themes. These themes included five patient centred themes (health risks, diverse sex acts, painful vaginal intercourse, relationship breakdown and violence, unwanted sex) and six health practitioner centred themes (communication and counselling, what is normal, ongoing care and follow up, emotional response, limited practical training, and highly prevalent). Participants described a complex sexual health landscape, with social contexts impacting women’s sexual experiences and engagement in treatment. Additionally, health practitioners reported the need for a biopsychosocial approach to understanding and responding to unpleasant sexual experiences for patients, while simultaneously reporting limited education in this area. Findings reflect the need for health practitioners to be cognisant of matters related to sexual function, consent, coercion, client engagement, and treatment pathways, identifying a need for greater education and holistic approaches to sexual healthcare across medical settings.

## Introduction

Sexual health and well-being, including sexual pleasure, are important to overall health and well-being (Ford et al., 2019). People who report more pleasurable sexual experiences report better overall sexual health, and this is more pronounced for women than men (Klein et al., 2022). The World Association for Sexual Health (WAS) Declaration of Sexual Rights asserts that sexual rights are fundamental to health; all people have the right to seek, receive, and impart information regarding sexuality to support well-being in healthcare settings (Ford et al., 2019; Kismödi et al., 2017; WAS, 2014). Despite the importance of sexual well-being, research into Australian sexual well-being in 2012 found that 48% of men and 68% of women report at least one sexual difficulty (Richters et al., 2022), and broader global research into sexual pleasure consistently finds that there is a substantial gendered gap in sexual pleasure between men and women. Women experience significantly less sexual pleasure than men, with heterosexual women being most impacted (Conley & Klein, 2022; Frederick et al., 2018; Laan et al., 2021; Richters et al., 2022). This is exemplified by the “orgasm gap,” where heterosexual women report orgasms in 65% of sexual encounters, compared to heterosexual men in 95% of encounters (Frederick et al., 2018).

It is well understood that non-consensual sexual encounters are inherently unpleasant and significantly impactful for men and women, as are unpleasant sexual encounters resulting from medical conditions, however, unpleasant sexual experiences also occur within chosen sexual activities in the absence of medical illness (Chadwick et al., 2019). Research into human sexuality indicates that people engage in a diverse range of sexual behavior as part of sexual expression (Herbenick et al., 2017, 2020), and that patterns of sexual behavior change over time alongside changes in social attitudes (Herbenick et al., 2022a, 2022b, 2022c). Sexual activities that were once considered “taboo” are becoming more prevalent, and men and women are increasingly engaging in sexual acts such as anal sex, sexual choking (as a form of asphyxiation or strangulation), and rough sex as part of their sexual repertoire (Herbenick et al., 2017, 2021, 2022a, 2022b, 2022c, 2023; McBride & Fortenberry, 2010; Rissel et al., 2014; Sharman et al., 2024). While not always wanted or desirable, people may engage in sexual acts (and endure associated discomfort) due to coercion or to please their partner (Carter et al., 2019; Fahs & Gonzalez, 2014; Jozkowski & Peterson, 2013; Marston & Lewis, 2014; Reynolds et al., 2015). Women are more likely to experience this pressure, and about 50% of women do not tell their partner if they are experiencing pain or discomfort during sex (Carter et al., 2019).

An estimated 37% of women and 9% of men report receiving anal sex across their lifespan (Herbenick et al., 2015). Despite this, only 24% of men and 14% of women report anal sex as appealing, and 72% of women and 15% of men report pain during anal sex (Herbenick et al., 2015). Anal sex carries health risks such as heightened risk of STD transmission, abrasions, fissures, bleeding and is associated with higher rates of faecal incontinence (Hutton et al., 2013; Markland et al., 2016). Rough sex, which can include whipping and spanking, bondage, hair pulling, verbal aggression or degradation and choking, is also prevalent, rated as appealing by approximately 40% of men and women (Herbenick et al., 2017). Rough sex acts are considered gendered, where women are predominantly the target of rough sex acts and men take a dominant role. For example, in a nationally representative US sample (*N* = 2,227, 51.73% female), one-fifth of women reported having been choked during sexual activity (Herbenick et al., 2023). Some research has linked the prevalence of rough sex to exposure to violent or aggressive sexual acts in pornography (Herbenick et al., 2021, 2023; Vogels & O'Sullivan, 2019; Wright et al., 2022).

There are risks associated with any rough physical acts; however, “choking” carries particularly heightened risks due to the restriction of blood flow and/or airflow which include, headaches, loss of consciousness, stroke, seizures, and even death (Bichard et al., 2022; Herbenick et al., 2022a, 2022b, 2022c; Wright et al., 2022; Yardley, 2021). Strangulation risks are increased, as it is indicated that many people do not research harm reduction strategies before engaging in sexual “choking” (Herbenick et al., 2022a, 2022b, 2022c). The variation and normalisation of diverse sexual acts involves physical and emotional health risks, and may be contributing to unpleasant, unsafe, or undesired sexual experiences for men and women. In understanding sexual pleasure as it relates to well-being, it is important to consider the prevalence of unpleasant sexual experiences, and the public health response.

While research into help-seeking for difficult/unpleasant sexual experiences is sparse, information on help-seeking for perceived sexual dysfunction provides some insight into people’s willingness to engage in sexual health care. Similar to unpleasant sexual experiences, the issue of sexual dysfunction is often unreported, unrecognised, and untreated in healthcare settings, despite being associated with poor physical health outcomes and psychological distress (Azar et al., 2013; Parish et al., 2019; Richters et al., 2022). Global rates of help-seeking for people experiencing sexual dysfunction indicate less than 25% of people seek treatment from a healthcare practitioner, including general practitioners, gynaecologists, psychologists, and sex therapists (Lafortune et al., 2023; Moreira et al., 2005). Barriers to help seeking include; shame or embarrassment, identification of sexual dysfunction as a medical issue, awareness of professional support available, the availability of health resources, direct questioning from health practitioners, and the effectiveness of health practitioner responses (Azar et al., 2013; Lafortune et al., 2023; Moreira et al., 2005; Shifren et al., 2009).

General practitioners are often considered the gatekeepers of the health system in Australia, with GPs being the primary source of referral to specialized health practitioners. This places GPs at the forefront of screening and treatment for sexual health and well-being concerns. Health care practitioners predominantly believe that it is important to discuss sexuality and sexual well-being with patients (Dyer & das Nair, 2013; Stott, 2013). Despite this, it indicated that 75–90% of people report health practitioners do not directly approach them about their sexual health or well-being (Wendt et al., 2007; Zakhari, 2009). GP’s report discussing sexual well-being with clients to be problematic and complex (Gott et al., 2004; Stott, 2013; Tarzia et al., 2019), indicating that sexual health and well-being is overlooked in primary healthcare settings. Previous research into health practitioner perspectives has suggested several reasons for this, including; limited resources, limited training, low confidence, concerns about offending the patient, limited awareness of sexual issues, and personal discomfort (Byrne & Sharman, 2022; Dyer & das Nair, 2013; Gott et al., 2004). International reviews indicate that GPs and other healthcare practitioners receive limited core training in sexual health, and that they are required to seek independent professional development post-graduation if they wish to increase their knowledge (Mollen et al., 2018; Stott, 2013; Wakley, 2000).

Despite sexual and reproductive health being considered a priority area in the National Women’s and Men’s Health Strategies 2020–2023, Australian health strategies and policies maintain a strong focus on reproductive health and sexually transmitted infection prevention, with limited focus on sexual function and well-being (with the exception of male erectile dysfunction) (Commonwealth of Australia, 2019a, 2019b). There is limited research into health care professionals’ experiences and training in treating sexual health and well-being in an Australian context. Similarly, there is a dearth of research on Australian healthcare practitioners’ experiences, attitudes, and confidence in treating or responding to clients unpleasant or uncomfortable sexual experiences. Given the prevalence and health implications of these experiences for both men and women, this study aims to add valuable insight by exploring health practitioners’ experiences with clients reporting unpleasant sexual experiences, by answering three key research questions: (1) health practitioners' experiences with patients who present with concerns related to unpleasant sex; (2) how are health practitioners responding; and (3) how prepared do health practitioners feel in assessing and responding to sexual health concerns from their patients?

## Method

### Participants and Procedure

Recruitment was facilitated by the Health Ed network who distributed the survey to their professional membership. The survey was also distributed via a snowballing approach, where a range of organisations, health services, and public health networks in North and South Brisbane were invited to distribute the survey to professional members. The survey was also distributed through social media groups, such as LinkedIn. Participation was voluntary and open to registered health practitioners in Australia. Data collection and informed consent were obtained using Qualtrics, and consenting participants completed the survey. Participants were asked to respond to demographic questions, then participants who reported experience in treating patients reporting unpleasant and/or painful sexual experiences were provided further questions related to their experiences. Due to the subjective nature of the term, “unpleasant sex” was defined as any sexual activity patients chose to engage in that was an unpleasant or uncomfortable experience for them, and examples were provided. Non-consensual sexual experiences (e.g., sexual assault) were excluded from this definition. Participant responses were anonymous, and assigned a unique identifier. The responses were analyzed using NVivo14 (2023) and quantitative data was provided by Qualtrics Survey Software.

A total of 171 participants consented to and commenced the study. Of these, 125 reported treating patients who had experienced unpleasant sexual experiences and 46 reported having no experience treating unpleasant sexual experiences. Of the 171 initial participants, ages ranged from 22 to 79 years (*M* = 51.86, *SD* = 12.91) and years of professional experience ranged from 1 to 55 years (*M* = 24.57, *SD* = 13.98). Participants without experience in treating unpleasant sexual experiences were then removed (*n* = 46), and participants who did not respond to further questions were removed (*n* = 29), leaving a final sample of 96 participants, 90 of whom completed all questions. Participants (*n* = 6) who provided partial responses, including demographic data, were retained in the sample to explore themes in their responses. Of the final sample of participants, ages ranged from 22 to 79 years (*M* = 51.74, *SD* = 12.63) and years of professional experience ranged from 1 to 50 years of experience (*M* = 24.95, *SD* = 13.15). All participants were located in Australia. Demographic data for these participants is reported in Table 1.

**Table 1:** Demographics of participants

Variables	*n*	%
*Sex*		
Female	78	81.25
Male	18	18.75
*Profession*		
General practitioner (medical doctor/physician)	62	64.58
Pharmacist	10	10.42
Nurse practitioner	6	6.25
Registered nurse	4	4.12
Registered nurse and midwife	4	4.12
Gynaecologist	4	4.12
Psychologist	2	2.08
Midwife	1	1.04
Clinical pharmacist	1	1.04
Sexual health physician	1	1.04
Unidentified	1	1.04
*Practicing state*		
New South Wales	33	34.38
Queensland	31	33.33
Victoria	14	14.58
South Australia	12	12.50
Tasmania	2	2.08
Australian Capital Territory	1	1.04
Northern Territory	1	1.04
Western Australia	2	2.08
*Location of practice*		
Urban	55	57.29
Outer metropolitan	21	21.88
Rural	18	18.75
Remote	2	2.08

### Measures

This study employed a mixed-methods quantitative and qualitative design, using an online survey with 25 questions, utilizing a cross-sectional online approach. Quantitative questions were used to obtain demographic information, prevalence data, and practice information, while open-ended qualitative questions were used to gather participant-led information on the subjective experiences of health practitioners and their conceptualization and treatment of patients presenting with unpleasant sexual experiences.

An online survey was administered using Qualtrics Survey Software. Guided by Simonis et al. (2016), two general practitioners (one with a specific interest and research base in sexual health) and both affiliated in research positions within an Australian University were consulted to assist in formulating survey questions. Demographic questions included: age, identified gender, profession, and practicing location. Qualitative open-ended and quantitative Likert-scale and dichotomous questions were utilised to gather feedback on health practitioners’ experiences. Qualitative questions explored health practitioners experience of client presentations, conversations with clients about unpleasant sexual experiences, perceived risks of unpleasant sexual experiences, and treatment approaches (e.g., “What kinds of problems related to unpleasant sex do your patients/clients often present with?”). Quantitative questions ascertained perceived prevalence and demographics of clients presenting with unpleasant sexual experiences, confidence in responding to patients in these settings, and training in supporting, treating, or responding to unpleasant sexual experiences (e.g., “How confident are you in answering questions from patients/clients regarding painful or unpleasant sex?”). See Appendix 1 for full survey questions.

The qualitative methodology for this research involved thematic analysis and took a critical realism ontological stance with a subjective epistemology, applying a constructivism paradigm. This approach allows for the acknowledgement and exploration of individuals lived experiences and how the interpretation and contextualisation of these experiences within broader social and cultural contexts makes meaning for the individual, allowing understanding of individuals subjective realities. This method supports developing an understanding of the experiences of the health practitioners who participated, reflecting their subjective and contextual perspectives and interpretations and how these are influenced by broad sociocultural contexts.

### Data Analytic Plan

Qualtrics Survey Software provided analysis of quantitative data and generated descriptive statistics including mean, standard deviation, range, and percentages. Qualitative data were transferred from the survey and analysed using NVivo (Lumivero, 2023), following Braun and Clarke’s (2013) six phases of thematic analysis. The analysis involved familiarization with the data (e.g., repetitive reading), generating codes relevant to the research questions and content similarities, and collating codes into patterns (i.e., potential themes). Authors RS, AA, and AC worked through these processes independently. The authoring team then collaboratively reviewed and refined potential themes across several meetings, conceptualising categories and subcategories. Thematic findings were discussed until discrepancies in interpretation reached a consensus, with minimal differences in interpretation between the authors. While the use of data extraction through online, open-response questions may have reduced the risk of interviewer bias, bias still exists in the thematic analysis process. Accordingly, it is worth acknowledging that the authoring team, two female-identifying psychology students (KvN and AC), one female-identifying academic (RS), one male-identifying academic and clinical psychologist (AA), one female-identifying general practitioner, and one male-identifying general practitioner may collectively have personal experiences that influenced the interpretation and analysis of the data.

## Results

### Thematic Analysis

Eleven themes with several subthemes were generated (see Table 2), and are presented in order of prominence. Descriptive results indicated that most participants saw patients presenting with difficulties arising from unpleasant sex once every three months (28.1%) followed by once a month (22.9%), once year (22.9%), once every two weeks (15.6%), once a week (8.4%), and daily (2.1%). The majority of participants reflected that they hear about unpleasant sex from patients when they aren’t presenting for specific advice regarding unpleasant sex sometimes (52.1%), followed by rarely (35.4%), often (10.4%), and very often (2.1%). Most participants reported that patients were predominantly women, and only four comments related to male-specific issues (e.g., erectile dysfunction), which were not analysed further as it was not clear if this related to cisgender males or transgender women. Other descriptive statistics are mapped onto and discussed alongside developed themes.

**Table 2:** Themes and subthemes of health practitioners treatment of unpleasant sex

Theme	Subtheme and Example Quote
Health Risks	*Physical health risks* “Risks to life or bodily safety or physical health depending on the activity” (P19) *Mental health risks* “Depression and other mental health effects including PTSD. Aversion to all sexual activity” (P15)
Diverse sex acts	“Oral sex, anal sex, sex in public places, being choked during intercourse” (P7)
Painful vaginal Intercourse	“Pain with penetration. Nil enjoyment of having sex because of the upcoming pain.” (P40)
Relationship breakdown and violence	“Pushing the partner away, some people are incapable of having intimacy without it leading to sex, this can lead to a complete breakdown in relationships” (P70)
Unwanted sex	“Feeling pressured to have sex with their partners when they really don't feel like it” (P68)
Communication and counselling	“It is part of the spectrum of human experiences with which people present to their general practitioners. I listen and I explore what is happening for them in a non-judgemental way.” (P80)
What is Normal?	“Some [patients] have [the] impression that painful sex is normal and expected and if it’s not painful then they are somewhat promiscuous” (P26)
Ongoing care and follow up	“Talking and exploring to assess if I’m the right person to continue discussions, or referral” (P16)
Emotional response	“I feel upset and sometimes at a loss of what to suggest” (P79)
Limited practical training	“I had zero undergraduate training in this aspect of sexual health. As I have done many years of training and practising in women’s health, I now feel more confident, but I do believe that undergrads should have training.” (P42)
Highly Prevalent	“[It’s] much more prevalent in society then seen/reported, not enough public awareness” (P22)

### Health Risks

Participants identified several health risks associated with unpleasant sexual experiences, resulting in two subthemes: physical health and mental health. Physical health emerged as the most prominent theme. Participants stated that patients discuss unpleasant sexual experiences in the context of general and sexual health checks. Participants identified that health risks could be short-term (such as tears or bruising) but that there were substantial risks of patients developing long-term health conditions (such as infections or internal scarring). One participant explained the aetiology of more serious conditions from minor injuries, stating “tearing of delicate tissue resulting in bleeding and infection of bladder” (P37) was a concern, and another identifying the risk of “long term fertility issues related to trauma or infection” (P40).

Mental health was the second reported theme regarding health risk, with participants identifying that unpleasant sex posed risks to well-being, causing distress, shame, and confusion, but also contributing to pervasive mental illness such as depression and PTSD. One participant cited “depression, anxiety, self-harm [and] in the extreme cases suicide” (P68) as potential outcomes. It was further identified that declined mental health may place patients at risk of ongoing unpleasant sex due to the “risk of developing an unintended attitude around what healthy safe sex actually constitutes” (P69), creating a cycle defined by one participant as “painful sex [leading to] anxiety [leading to] tension [leading to] painful sex” (P47).

### Diverse Sex Acts

According to descriptions from participants, unpleasant sexual experiences were characterised by a range of sex acts disclosed by patients, distinct from ‘traditional’ vaginal sex. Anal sex was frequently mentioned, with participants highlighting anal sex as an act that can be considered both physically and emotionally unpleasant. This was captured by one participant who identified patients experiencing “anal sex [as] painful, degrading and unpleasant” (P25). The second most commonly discussed sexual experience was that of “rough sex,” reported by participants to include: rough penetration, choking, hitting, biting and physical restraint. One participant reflected that patients experience “rough sex when it’s really violent sex” (P70), highlighting the diverse intensity of “rough” and the apparent blurred line that may exist between a sexual act and violent one. Oral sex, public sex and group sex were also discussed, though less frequently, as unpleasant sexual experiences.

### Painful Vaginal Intercourse

Participants noted painful vaginal intercourse as a common issue that patients present with. Responses reflected diverse aetiologies of pain including vaginal dryness or poor lubrication, latex allergies, uncomfortable positions, partner penis size, and recent physical trauma (such as childbirth). One participant detailed the variance in presentations, stating they see patients for “pain during sex due to dry vagina, pain due to allergy from using condoms, pain due to refusing to have sex [and] pain after childbirth, especially if she had an episiotomy” (P83). Some participants referenced existing diagnoses as contributing to painful vaginal intercourse, such as endometriosis and vaginismus, with other conditions identified by one participant who stated, “the patients I see with painful intercourse have either provoked vestibulodynia or introital stenosis due to lichen sclerosus” (P60). Of relevance to this theme is the persistence of patients in engaging in painful vaginal sex, despite reporting pain and finding the experience unpleasant.

### Relationship Damage and Violence

Responses outlining relationship breakdowns and increased risks of violence were reported by participants. Participants identified that patients avoid or disengage from sex with their partner, or show less enjoyment during sex, following unpleasant sexual experiences, which can lead to reduced intimacy and conflict, straining partner relationships. One participant explained this pattern, stating it “may lead to stress in relationship and avoidance of sex [which] can lead to conflict and risk of sexual coercion” (P74) and another noted that patients’ experience “backlash from [their] sexual partner as [they’re] not seen to being enjoying sexual acts” (P44). Participant responses noted that patients’ partners may respond with sexual or financial coercion, abuse, or violence, and that patients may experience associated financial, social, and cultural consequences that reinforce patterns of coercive control, summarised by one participant as a “high risk for escalating domestic violence” (P55).

### Unwanted Sex

The theme of Unwanted Sex details participants reports of patients’ engaging in sex or sexual acts due to implicit and explicit pressure from their partner, despite actively disliking the act or finding it physically or emotionally uncomfortable. Content of this theme included: obligatory or duty sex, pressured sex and giving in, pleasing a partner and engaging in sex acts to seem “normal,” detailed in Table 3. Despite the study’s definition of unpleasant sex explicitly excluding non-consensual sex, participants still reported sexual assault, forced sex, and non-consensual removal of condoms as “unpleasant sex,” possibly reflecting disparate perceptions of consent. Participant responses to conversations related to consent are clarified in the theme: What Is Normal?

**Table 3:** Compliance with unwanted sex

Discourse	Example quote
Duty Sex	“Marriage sex….feeling forced to have sex with partner because or culture or marriage even if they don’t want to.” (P59)
Giving in	“‘Giving in’ to partner’s insistence for sex when they are disinterested, tired or not attracted to their partner. Performing sexual acts that their partner enjoys that they find embarrassing or humiliating” (P61)
Pleasing	“They want to feel part of the experience, they feel they are letting their partner down, so they just let them do what they want to keep them happy” (P21)
Normalizing	“Coercion to take part in activities which are unpleasant so that they are seen as 'normal'” (P74)

### Communication and Counselling

Communication and counselling was the most reported theme by health practitioners regarding their responses to patients presenting with unpleasant sexual experiences. This theme identifies that patients are not always cognisant of their experience as problematic, feel uncomfortable disclosing and are unaware of interventions. In responding to this, health practitioners aim to take a professional stance prioritising objectivity and non-judgement so that patients feel confident in practitioners’ ability to respond, and provide emotional support to patients to increase their comfort to discuss their experiences. This was perceived to facilitate a thorough understanding of the client’s history and develop treatment responses that are sensitive to client's needs. One participant advised their response was to “reassure them that you are comfortable to discuss this and also to work towards helping resolve the issues” (P74) and another reported “active listening and explor[ing] their concerns, providing [a] safe space to express their experiences and emotions” (P46).

### What Is Normal?

Sex and normality emerged as a theme identifying health practitioners’ role’s in communicating and affirming for patients what “normal” and acceptable sexual experiences are. Participants reported that patients seek advice from them to understand normal physical or emotional responses to sexual experiences, whether their experiences are normal and how to communicate boundaries and preferences to their partners. One participant outlined that patients are “wanting to confirm that they are not the only person who finds this activity unpleasant, wanting to normalise their concerns and discuss how to bring this up with partner” (P74). It was reported that patients may normalise unpleasant sexual experiences, based on repeated experiences, social feedback, or exposure to pornography. This was explained by one participant who stated:

Some present because their partners, who want sex several times a day, think they have a sexual dysfunction because they are ok with once per week. A lot of conversations are giving the woman permission to say no to the sex and explore alternative ways to be sexually intimate, and to normalize sexual disparities. (P70)

Participants identified that these perspectives can be challenged and that they take an active role in providing reassurance, education, and explicit coaching to patients across domains of sexual functioning, including physiological sexual responses, consent, and communicating sexual preferences and boundaries. One participant detailed they have learned to take proactive action, stating:

I often talk with younger female patients presenting for contraception about consent and the impact of pornography on sexual acts and body dysmorphia—as a lot of issues that patients present with tend to be related to being coerced into unpleasant sex that their (usually young male partner) has learned to be 'normal' through pornography. (P40)

Participants reported that they actively check for issues regarding consent or intimate partner violence, as outlined by one participant stating, “I would first screen for patient safety i.e., is this “merely” unpleasant, or is it coercive/nonconsensual? Is there an issue generally within the relationship that would compromise patient safety such as controlling or abusive behavior” (P61).

#### Ongoing Follow Up and Care

**Fig. 1:**
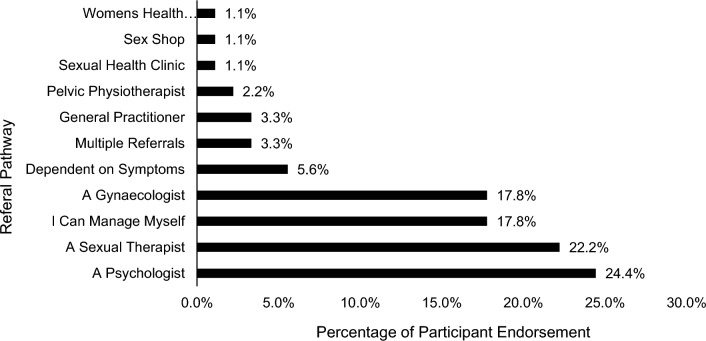
Referral pathways *Note* Total responses *n* = 90

This theme emerged through participant reports of ongoing care for clients, including follow-up and referral for additional support as needed. Ongoing care was considered case-specific, requiring targeted interventions to meet the client’s specific presentation and needs. It was identified that referral is an important aspect of ongoing care, with 82.2% of participants endorsing referral to other professionals, with endorsed referral pathways detailed in Fig. 1. Participants identified referral pathways as dependent on patient support needs, with one participant stating, “depending on the situation, sometimes referral to [a] psychologist is appropriate, sometimes patients/clients need to see a gynaecologist, a physiotherapist or a sexual therapist, or a team of health professionals” (P20). The prevalence of referral may be related to health practitioners’ perceived training and confidence, as explored in the theme Limited Practical Training.

#### Emotional Response

Participants reported a range of emotional responses regarding treating patients for unpleasant sex. Response data reflected that most participants felt comfortable (65.6%) or very comfortable (24.4%) advising patients about unpleasant sex, while a minority reported feeling not comfortable (10%) (*n* = 6 responses missing). Qualitative responses detailed emotional reflections from participants, including feelings of empathy, sadness, and concern. This was expressed by one participant as: “I feel sad for them, concerned [and] wanting to help” (P12). Participants reported that there was a sense of gratitude, relief, and honour that came with patient disclosures. One participant reported “I feel honoured that they trust me and believe in me enough to discuss this” (P42). Finally, some participants reported feelings of shock, discomfort, and overwhelm when discussing these experiences with patients, reporting a sense of ill-preparedness to respond. This was expressed by one participant as “I feel upset and sometimes at a loss of what to suggest” (P79).

#### Limited Practical Training

Low levels of practical training were a frequently endorsed theme. Overwhelmingly, 96.7% of participants reported receiving minimal undergraduate training related to unpleasant sex, while 1.1% reported adequate training and 2.2% reported comprehensive training (*n* = 6 responses missing). Slight improvements were noted regarding participants’ continued education, but the majority (72.2%) of participants continued to report minimal ongoing training, while 18.9% reported adequate training and 8.9% reported comprehensive training. Lack of professional training and the need for professional development were reflected in the qualitative data, with participants stating, “we need more training on this issue” (P33). Some participants reported sourcing their own training and relying on practical experience to support confident practice. Response data showed that roughly half of participants reported being completely confident (15.6%) or fairly confident (40%) in answering questions from patients in regard to unpleasant sex, with the remainder feeling somewhat confident (22.2%), slightly confident (18.9%), or not confident at all (3.3%), perhaps reflecting individual investment in professional education or learning.

#### High Prevalence Issue

While not as prevalent as other themes, and not directly queried in the survey, several participants provided responses reflecting the prevalence of unpleasant sexual experiences, noting it as both a significant and complex issue. This was reflected strongly by one participant stating, “this is a HUGE issue and especially in remote communities where I work” (P68). Participants reported the youngest patient age they had seen reporting unpleasant sexual experiences ranging from 12 to 55 years (*M* = 19.94 years, *SD* = 6.42) and the oldest patient ranging from 20 to 86 years (*M* = 63.34, *SD* = 13.94) reflecting a wide age range of patients needing treatment.

## Discussion

The present study aimed to investigate health practitioners’ experiences treating patients presenting with unpleasant sexual experiences, seeking to understand what health practitioners are facing, how they’re responding, and their preparedness to meet patients’ needs. Qualitative and quantitative responses resulted in 11 core themes. Throughout these themes, there was a consistent discourse reflective of the complexity of sexual health and key issues in providing a healthcare response.

### Experiences of Health Practitioners

Five themes emerged in response to the first research question: health risks, diverse sex acts, painful vaginal intercourse, relationship breakdown and violence, and unwanted sex. These themes were reflective of patients reports and health practitioners’ interpretations of significant social, emotional, and physical risks related to unpleasant sexual experiences. Reports of physical health risks as a prominent theme were unsurprising, and consistent with the body of existing research reflecting the health risks of unpleasant and painful sex acts (Bichard et al., 2022; Hutton et al., 2013; Markland et al., 2016; Yardley, 2021).

Our findings reflected that health practitioners view unpleasant sex through a gendered lens, reporting women of all ages as the primary group experiencing this issue. This view is consistent with existing research that identifies women experience less pleasant sex than men (Conley & Klein, 2022; Laan et al., 2021) and experience more painful sex than men (Carter et al., 2019). In addition to commentary on painful and unpleasant sex, the themes of relationship breakdown and violence and unwanted sex show health practitioners’ consideration of the interplay of psychosocial factors in unpleasant sex. This perspective is supported by current research regarding female sexual dysfunction, which highlights the medical, psychological and relational/social elements implicit in the aetiology and maintenance of unpleasant sex for women (Meana & Binik, 2022). This lens was similarly reflected by health practitioners view of diverse sex acts, such as anal sex and rough sex, showing consistency with prior research that demonstrates that women rate diverse sex acts as less appealing than men (Herbenick et al., 2017), but that women engage in diverse acts despite lack of interest or appeal (Faustino & Gavey, 2022) and are often the recipient of acts such as anal sex, choking, or hitting (Herbenick et al., 2021; Vogels & O'Sullivan, 2019; Wright et al., 2022).

Health practitioners’ commentary on unpleasant sex acts as a complex issue centred in gendered social contexts aligned with prior research on gendered sexual scripts, as reflected by Ward et al. (2022) and Wright et al. (2021), where western social ideologies, media, and pornography develop and reinforce sexual attitudes placing women in roles of submission and subservience in sexual encounters, resulting in lower sexual agency, prioritisation of partner pleasure (Curtin et al., 2011), and greater acceptance of male sexual aggression (Papp et al., 2021). Topics of consent and coercion were focal points throughout this study, with health practitioners identifying issues of sexual agency, partner-pleasing, and non-consent as central to women’s engagement in unpleasant and unwanted sexual experiences.

There is a breadth of literature identifying the complexities of consent and coercion in western society, with one prominent view being that social scripts position women as ‘gatekeepers’ to sex and men as initiators (Jozkowski & Peterson, 2013). Existing research suggests that men prioritise implicit, non-verbal indicators of consent, reflecting the need for women to provide active verbal assertion of non-consent to cease unwanted sexual activity (Kubota & Nakazawa, 2024), and that assumed consent is common in long-term relationships (Humphreys, 2007). The present study identified that barriers to assertive non-consent may be implicated in women’s experience of unpleasant sex due to women not wanting to upset their partner, internalising blame, and due to the identified risk of relationship breakdowns or escalating relational violence. The present study highlighted that health practitioners see patients engaging in unpleasant sexual experiences due perceived and overt partner pressures and experiencing associated negative outcomes such as pain, injury, and emotional harm. The observations of health practitioners may be understood through research on sexual coercion as predictive of sexual compliance both in the moment and over time (Katz & Tirone, 2010), and as a factor in women’s decisions to engage in unpleasant heterosexual anal sex (Fahs & Gonzalez, 2014; Faustino & Gavey, 2022) and choking (Herbenick et al., 2022a, 2022b, 2022c). Participants in this study referenced patients who sought permission to say ‘no’ to unpleasant sex acts, seeking an understanding of whether their dislike of a sexual experience was ‘normal’ or valid, or if the issue they were experiencing was their fault. These findings raise the importance of health practitioners having a comprehensive understanding of the wider social contexts that influence sexual health landscapes and practices, and the need to be vigilant of the risks of coercion, non-consent, and violence for participants reporting unpleasant sexual experiences.

### Health Practitioner Responses

The second research question queried how health practitioners approach treating unpleasant sexual experiences, with results outlining three themes of: Communication and Counselling, What is Normal, and Ongoing Care and Follow Up. Initially, the themes highlight that health practitioners navigate reports of unpleasant sex through professional and empathetic engagement. This practice approach is consistent with past research, identified as important in both sexual health and general health strategies (Larsen & Cecchini, 2023) and relates to previously reported views that establishing rapport is important, before responding to sexual health matters (Hendry et al., 2018). Contrary to previous research (Wendt et al., 2007), health practitioners in this study reported that they actively discuss sexuality and abuse with patients, directly exploring issues related to sexual health, consent, and interpersonal violence. It is noted that previous research has found that professional interest in sexual health and work in sexual health services may increase health practitioners engagement in these conversations (O'Sullivan et al., 2019), this study may have attracted practitioners meeting these criteria given by its distribution via a professional education network (HealthEd) or, alternatively, comfort may be increasing over time due to shifts in social values.

Secondly, this study identified health practitioner efforts to address the social and relational issues that contribute to patients’ experiencing unpleasant and unwanted sex, through taking an active role in educating patients in consent, communication, normal sexual function and promoting safety and agency. Health practitioners are generally perceived as an authority, where the unique insights they provide are heard and acted upon (Larsen & Cecchini, 2023). This positions health practitioners in good stead to educate and empower patients, and these conversations may be particularly pertinent for patient groups who have missed out on targeted sexual education, such as older generations (Graf & Johnson, 2021). In the absence of any clear framework to guide health practitioners in these conversations, there is the risk that personal values and perspectives on sex and consent may bias advice, with previous research indicating that personal values can dominate professional values in practice (Hendry et al., 2018; Muhamad et al., 2019). This indicates that patient experiences can be dependent on the treating practitioner’s values, comfortability, and professional knowledge, risking inconsistent outcomes for patients and justifying the need for practice guidelines in this space.

Finally, the present study found that health practitioners consider ongoing care as central to treatment for patients, citing various referral pathways for treatment and support, reflective of medical treatment recommendations such as psychology (Alahverdi et al., 2022; Mestre-Bach et al., 2022), physiotherapy, and sex therapy (Cacchioni & Wolkowitz, 2011). This was consistent with the overall endorsement of a holistic approach to understanding and responding to patients reporting unpleasant sexual experiences, seeing unpleasant sex through lenses of physical health, mental health, and social contexts, responsive to the complexity of the topic which is aligned with modern views of female sexual function (Meana & Binik, 2022; Rosen & Barsky, 2006).

### Preparedness to Respond

The final research question that this study aimed to answer was health practitioners perceived preparedness to meet the needs of patients reporting unpleasant sexual experiences. Three themes were identified in relation to this question: emotional response, limited practical training and highly prevalent. Despite viewing the issue as prevalent and important, the present study highlighted that health practitioners perceive substantial gaps in their undergraduate training regarding sexual health and unpleasant sexual experiences. This is consistent with previous qualitative research in Australia (Lucke, 2017) and overseas (Dyer & das Nair, 2013; Stott, 2013), identifying that lack of training is cited by a range of health practitioners as a barrier to both initiating and responding to conversations related to sexual health and well-being. Responses in this study found that post-graduate professional development in this area was not regularly obtained by participants, with under 25% of participants reporting intentional ongoing training.

Despite limited training, over 90% of participants in this study reported feeling comfortable discussing unpleasant sexual experiences with patients, and over 75% reported feeling confident providing advice. This was contrary to previous research, where health practitioners have routinely cited personal discomfort and the complexity of topic as a reason for not engaging with patients regarding sexual health (Dyer & das Nair, 2013; Gott et al., 2004). Regardless of comfort and confidence, health practitioners reported that discussions related to unpleasant sex carried an emotional weight, contributing to stress and sadness, but also satisfaction and pride. These findings present a professional landscape where health practitioners are trying to respond to a complex and prevalent issue, without the training to support them. This may be a pertinent consideration where diagnosis uncertainty, emotional exhaustion and job dissatisfaction are related to health practitioner’s high burnout rates (Zhou et al., 2022). While not reported on by participants in this study, considerations raised in previous research indicate that limited resources such as time, high caseloads and a shortfall in health practitioner numbers can make it more challenging for health practitioners to provide a thorough response to patients (Australian Medical Association, 2022).

### Implications

This study is the first of our knowledge to explore health practitioners’ experiences of treating patients presenting to medical settings for unpleasant sexual experiences, and is considered to have several strengths. The online sampling method provided substantial reach across Australia, reflecting diverse health contexts across rural and urban Australia, while limiting the risk of researcher bias. The large sample size provided an overview of the experiences of health practitioners.

While this study took an atheoretical approach, participant responses can be understood through the biopsychosocial theory of medicine (Engel, 1977) and feminist theories on the social roles and historic disadvantages impacting women. Health practitioners’ perspectives consistently reinforced the need to both interpret and respond to unpleasant sexual experiences from a biological, psychological, and sociological perspective, with awareness of how women are uniquely impacted by social contexts. This approach reflects a significant and positive change from historical approaches, which viewed female sexual dysfunction as it related to their role in sexually pleasing their partner or conceiving children, and described it as “frigidity” or “psychological disturbance” with treatment approaches including hypnosis, systemic desensitization, and “re-education” (Burdine et al., 1957; Popenoe, 1954; Sotile & Kilmann, 1977).

The present study has several implications in understanding the experiences of health practitioners in treating women experiencing unpleasant and unwanted sex, and in providing recommendations to improve the capacity of health practitioners to respond and improve the experience of patients. The 11 themes identified in this study, alongside existing research, reflect a complex sexual health landscape that is dominated by social issues related to women’s sexual agency, such as gendered social scripts, consent education and coercion. It is indicated that health practitioners are well placed to provide education, empowerment, and treatment for patients when they have sufficient training, feel confident and comfortable and hold values consistent with best practice. These findings reinforce the need for in-depth practical training and practice guidelines for health practitioners, encompassing holistic approaches to sexual health, the psychological and social contexts that impact women’s sexual experiences, and ways to engage patients in these discussions to increase disclosure and therefore access to treatment. Health practitioners may find the findings of this study of interest, as it relates to how patients may present and ways to engage and support patients.

### Limitations and Future Directions

Whilst the use of the term “unpleasant” sex was chosen specifically as a bridge between normative, subclinical, and pathological aspects of sexual activity, such a broad term is encompassing and may not have allowed for clear specificity in responses. Similarly, while the research method allowed for diverse sampling, it did limit the ability to seek clarity or elaborate on responses, which may have contributed to misinterpretations of participant responses and impacted the richness of data. As the research was situated in Australia, and the survey primarily distributed via a professional network, there are limitations to cross-cultural generalisability. Further to this, as the sample of participants was over 80% women, the results may reflect health practitioners with particularly interest or investment in female sexual health, perhaps biasing the results and limiting generalisability. As our research focused on health practitioners’ individual experiences, further research into training approaches for health practitioners may be beneficial, alongside research into screening approaches for patients to better identify and respond to unpleasant sexual experiences and the development of practice guidelines to support a best-practice approach to sexual healthcare for women experiencing unpleasant, unwanted, and painful sex.

### Conclusion

In conclusion, health practitioners across a suite of professions are seeing patients reporting unpleasant sexual experiences, situated in complex social dynamics related to women’s sexuality, requiring a biopsychosocial response to exploration, engagement, and treatment. Health practitioners are the forefront of identification, diagnosis, and treatment for sexual function, and are well placed to provide holistic care to patients reporting unpleasant sexual experiences. This is supported when practitioners are educated, confident and aware of the biopsychosocial contributors to unpleasant sex, but it is identified that they experience barriers related to training, patient comfort in disclosing, and unclear guidelines around responding to the issue. This is an important area of healthcare research, identified as a prevalent and complex topic requiring further investment.

## Data Availability

Data can be made available upon reasonable request.
